# Chemical Synthesis and Characterization of an Equinatoxin II(1–85) Analogue

**DOI:** 10.3390/molecules22040559

**Published:** 2017-03-30

**Authors:** John A. Karas, Marc-Antoine Sani, Frances Separovic

**Affiliations:** School of Chemistry, Bio21 Institute, University of Melbourne, Melbourne, VIC 3010, Australia; jkaras@unimelb.edu.au (J.A.K.); msani@unimelb.edu.au (M.-A.S.)

**Keywords:** native chemical ligation, solid-phase peptide synthesis, selectively labelled proteins, membrane protein structure, solid-state NMR

## Abstract

The chemical synthesis of an 85 residue analogue of the pore-forming protein, Equinatoxin II (EqtII), was achieved. Peptide precursors with over 40 residues were assembled by solid phase synthesis. The EqtII(1–46) fragment was modified to the reactive C-terminal thioester and native chemical ligation was performed with the A47C mutated EqtII(47–85) peptide to form the EqtII(1–85) analogue. Circular dichroism spectroscopy indicated that the N-terminal domain of EqtII(1–46) and EqtII(1–85) maintains predominantly an α-helical structure in solution and also in the presence of lipid micelles. This demonstrates the feasibility of assembling the full 179 residue protein EqtII via chemical means. Site-specific isotopic labels could be incorporated for structural studies in membranes by solid-state NMR spectroscopy.

## 1. Introduction

Equinatoxin II (EqtII), from the sea anemone *Actinia equina* is a 179 residue pore-forming toxin (PFT) and member of the actinoporin family [1,2]. According to nuclear magnetic resonance (NMR) and X-ray crystallographic data, it consists of a β-sheet core with a flexible α-helical N-terminal domain [3]. The mechanism of pore formation is well understood and proceeds via a number of distinct stages. Firstly, EqtII monomers bind to cell surfaces via the interaction of aromatic residues with sphingomyelin (SM) rich membranes [4]. The N-terminal α-helix then inserts into the membrane, followed by oligomerization and cell permeabilization [5,6]. There is evidence that the size of the pores that are formed are heterogeneous, as determined by conductance measurements. It is estimated that average pore consists of 3–4 monomers although higher oligomers are also possible [7]. However, the structure of the pore is not well characterized, such as the nature of the oligomeric interface between monomers and how lipids are involved in its architecture [5]. Therefore, alternative techniques need to be employed to aid in elucidating the mechanism of action of EqtII in SM rich membranes.

Solid-state NMR spectroscopy is a powerful tool for observing the molecular dynamics of proteins in membranes [8,9]. This technique is often enhanced by incorporating “NMR-active” nuclei such as carbon-13 and nitrogen-15 into a biologically expressed product [4], which significantly enhances the signal and simplifies interpretation of the spectra. This is typically achieved by spiking the protein expression media with isotopically enriched amino acids or precursor molecules such as glucose. Although this global labelling strategy is often effective, it is not optimal when analysis of a specific protein domain is desired. In these situations, site-specific labelling of key residues can be more advantageous [10]. Our long-term goal is to chemically synthesize specifically labelled carbon-13 and nitrogen-15 labelled EqtII analogues for 2D NMR experiments in model membranes [11,12]. This will allow for the incorporation of isotopically enriched amino acids at peripheral residues to detect intermolecular contacts between monomers. However, the chemical synthesis of ~20 kDa proteins is extremely challenging. Therefore, the scope of this work will involve the design and assembly of an EqtII(1–85) analogue from two synthetic peptide fragments, to validate our strategy.

**Figure 1:**
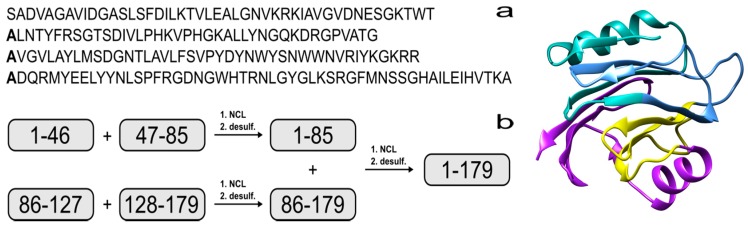
(**a**) Amino acid sequence of Equinatoxin II (EqtII). The alanine residues to be incorporated as cysteine are in bold; (**b**) Proposed strategy for the chemical assembly of native EqtII. Note that the scope of the work reported here involves the native chemical ligation (NCL) of the EqtII(1–46) and EqtII(47–85) to give the EqtII(1–85) analogue. The crystal structure of full-length EqtII (PDB: 1IAZ) is shown on the right.

The synthesis of proteins via expressed protein ligation (EPL) [13] or native chemical ligation (NCL) are well-established techniques [14,15] and more recently, proteins with 166, 228 and 312 residues have been assembled using NCL [16,17,18]. In brief, a peptide that possesses a C-terminal thioester can react with a second peptide that contains an N-terminal cysteine to form a native amide bond via thiol exchange and intramolecular rearrangement. Recent reports have shown that NCL can be achieved via 9-fluorenylmethoxycarbonyl solid-phase peptide synthesis (Fmoc SPPS) by using a C-terminal hydrazide as a thioester surrogate [19,20]. There have also been advances in peptide production, such as microwave-assisted Fmoc SPPS [21]. This technology enables the efficient assembly of longer peptides [22,23,24], which minimizes the number of steps required for NCL. Unfortunately, EqtII does not contain any cysteines that are necessary for thioester formation, but this residue can be incorporated in strategic positions as a surrogate for alanine to facilitate the ligation reaction. Native alanine residues are then regenerated via radical-initiated homolysis of the sulfur atom [25]. Using this strategy would make it feasible to produce EqtII from four fragments that range from 39 to 52 residues (Figure 1).

## 2. Results and Discussion

**Figure 2:**
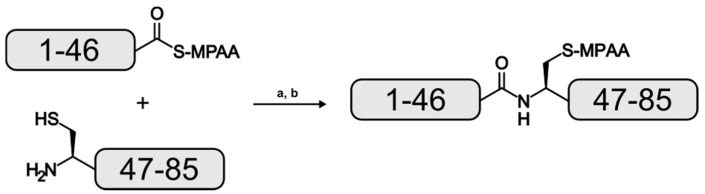
Reaction scheme for the formation of the EqtII(1–85) analogue. (**a**): pH 7, 20 °C, 18 h; (**b**): reversed-phase high performance liquid chromatography (RP-HPLC).

For this study, EqtII fragments 1–46 and 47–85 were isolated as C-terminal hydrazides with the latter possessing an A47C mutation. The 3-Methylpentyl protection was used for the β-carboxylate of aspartate residues instead of *tert–*butyl to minimize aspartimide formation and its associated side products [26]. Purified 1–46 was oxidized to the acyl azide which facilitated the formation of the reactive 4-mercaptophenylacetic acid (MPAA) thioester followed by a second purification [19]. An equimolar amount of purified 47–85 was added to the reaction mixture and the ligation was performed at neutral pH (Figure 2).

**Figure 3:**
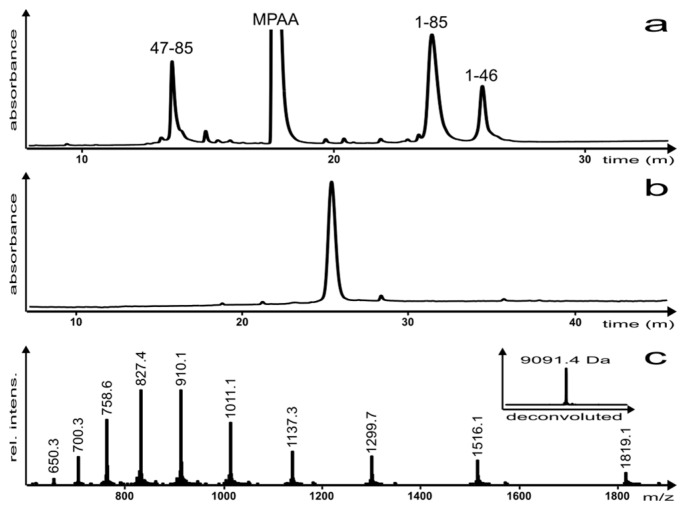
(**a**) NCL reaction after 4 h; (**b**) analytical RP-HPLC of the purified EqtII(1–85) analogue and (**c**) ESI-MS of the purified EqtII(1–85) analogue (inset: Deconvoluted spectrum; molecular mass (calc.) = 9091.4 Da). Please note that chromatograms (**a**,**b**) were analyzed using different HPLC instruments, which accounts for the small difference in the retention time of the EqtII(1–85) analogue.

The reaction was monitored by reversed-phase high performance liquid chromatography (RP-HPLC) (Figure 3a) and the product was characterized via analytical RP-HPLC and electro-spray ionization mass spectrometry (ESI-MS) (Figure 3b,c, respectively). Once the reaction was complete, the EqtII(1–85) C-terminal hydrazide analogue was isolated as the A47C disulfide-linked MPAA adduct (which can be reductively cleaved and desulfurized in a subsequent step) in a yield of 12%.

**Figure 4:**
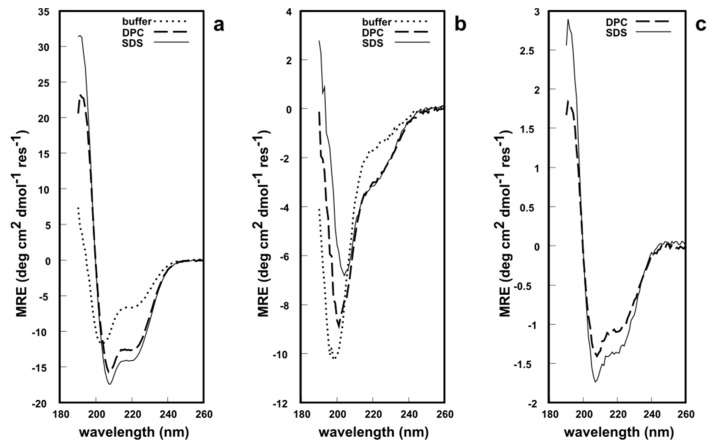
Circular dichroism (CD) spectroscopy of the: (**a**) EqtII(1–46) fragment; (**b**) EqtII(47–85) A47C fragment; (**c**) EqtII(1–85) fragment (as the 4-mercaptophenylacetic acid (MPAA) adduct). ~40 μM peptide/protein in buffer, 15 mM dodecylphosphocholine (DPC) or sodium dodecyl sulfate (SDS) micelles, pH 7.2, 25 °C.

All three fragments were analyzed via circular dichroism (CD) spectroscopy in phosphate buffer at pH 7.2, to determine whether these synthetic analogues possess a secondary structure similar to native EqtII. The CD lineshape of EqtII(1–46) in solution showed typical features of an α-helical structure with minima at 222 nm and 209 nm and a maximum at ca. 190 nm (Figure 4a). This is expected since the N-terminal domain can readily translocate and, therefore, does not require any structural constraints to maintain its native conformation. NMR and CD studies of the N-terminus of EqtII report an α-helical structure [27,28,29]. In the presence of either dodecylphosphocholine (DPC) or sodium dodecyl sulfate (SDS) micelles, the intensity of the minima and maximum increased (Figure 4a), which indicates a greater helicity of the peptides. By contrast, EqtII(47–85) in solution had a CD lineshape with a single minimum at ca 195 nm that is typical of random coil structures (Figure 4b). There is no β-sheet formation exclusively within this fragment in native EqtII according to X-ray crystallographic data [3], so this peptide, untethered from the native protein, would be unlikely to adopt a highly ordered conformation.

In the presence of DPC or SDS micelles, however, the CD spectra showed a reduction and a red shift of the minimum at 195 nm to 209 nm, while a second minimum at ~222 nm appeared, which indicates a significant increase in α-helical content. EqtII(1–85) was insoluble in the phosphate buffer and, therefore, a CD spectrum was not recorded. The fragment was soluble in the presence of DPC and SDS micelles (Figure 4c) and appeared to possess the expected α-helicity in both systems, given that it contains the N-terminal domain. In short, all CD spectroscopic data supports the notion that these chemically synthesized peptide fragments adopt a predictable secondary structure in solution (or lack thereof). EqtII(1–46) and EqtII(1–85) also appear to exhibit behavior similar to the native protein in the presence of membrane systems. The ^1^H solution NMR spectra of EqtII(1–46), EqtII(47–85) and EqtII(1–85) in DPC micelles exhibited ^1^H chemical shift dispersions and sharp resonances, supporting high helical contents (See Supplementary Material S1). Finally, these results with EqtII(1–85) indicate that full length EqtII(1–179) may be chemically synthesized and likely adopt the correct conformation.

## 3. Experimental Section

### 3.1. Materials

Peptide synthesis was performed on a CEM Liberty microwave peptide synthesizer (CEM Corp., Matthews, NC, USA). Standard Fmoc amino acids and Oxyma Pure were also purchased from CEM. Fmoc-Asp(OMpe)-OH, Boc-Gly-OH and 2-Chlorotrityl chloride resin (sub: 1.0 mmol/g) were obtained from GL Biochem (Shanghai, China). Diisopropylcarbodiimide, guanidine hydrochloride (Gn.HCl), diethyl ether, acetonitrile, dimethylformamide (DMF) and dichloromethane were sourced from Merck (Darmstadt, Germany). Sodium nitrite, sodium hydrogen phosphate (Na_2_HPO_4_), hydrazine, diisopropylethylamine (DIEA), piperidine, trifluoroacetic acid (TFA), triisopropylsilane (TIPS), 3,6-dioxa-1,8-octanedithiol (DODT) and MPAA were obtained from Sigma (Sydney, Australia). Dodecyl phosphocholine (DPC) and sodium dodecyl sulfate (SDS) were obtained from Sigma (Australia).

### 3.2. Peptide Synthesis

The peptide fragments were isolated as C-terminal hydrazides using standard microwave-assisted Fmoc-SPPS protocols. Briefly, 2-chlorotrityl chloride resin (0.3 mmol) was treated with excess hydrazine and 5 eq. of DIEA, followed by peptide assembly. The first amino acid was spiked with 2 eq. of Boc-Gly-OH to reduce the resin loading. All amino acids were coupled once at 90 °C except for arginine which was coupled twice and histidine which was coupled at 50 °C. Deprotection was performed with 20% piperidine in DMF and 0.1 M Oxyma at either 75 °C or ambient temperature (when an aspartate residue was present). Cleavage and global deprotection was performed with a cocktail of TIPS/H_2_O/DODT/TFA (2%/2%/1%/95%), followed by ether precipitation and RP-HPLC purification. The C-terminus of the N-terminal fragment was converted to the MPAA thioester via a previously reported method [19], followed by a second purification.

### 3.3. Purification and Analysis

HPLC analysis and purification were performed on an Agilent 1100 system (Santa Clara, CA, USA). Mobile phases: (1) buffer A = 0.1% TFA in water; buffer B = 0.1% TFA in acetonitrile. Purification was carried out on a Phenomenex Kinetex 5 µm XB-C18, 100 Å, 150 mm × 21.2 mm AXIA packed column. Typical gradient: 10–70% buffer B over 60 min with a 5 mL/min flow rate and detection at 230 nm. Analysis was performed on a Phenomenex 5 µm C18, 100 Å, 150 mm × 4.6 mm column. Typical gradient: 0–80% buffer B over 40 min with a 1 mL/min flow rate and detection at 220 nm. Mass spectrometry was performed on an Agilent 6510 ESI-TOF instrument. All peptides were lyophilized on a Virtis freeze dryer.

### 3.4. Native Chemical Ligation

EqtII(1–46) thioester (3.0 mg, 1 eq.) and EqtII(47–85) A47C (2.7 mg, 1 eq.) were dissolved in a solution of 6 M Gn.HCl and 0.2 M Na_2_HPO_4_ at pH 7 with MPAA (4.5 mg, 50 eq.). The reaction was stirred for 18 h at 20 °C, followed by purification and lyophilization. An amount of 0.6 mg of purified material was recovered, indicating a yield of 12%. Refer to Figure 3b,c for analytical data.

### 3.5. CD Spectroscopy

Detergent-free peptide solutions were dissolved in 10 mM phosphate buffer solution (NaCl 1 mM, pH 7.4) to a final concentration of ~40 μM. Appropriate aliquot of 200 mM DPC or SDS micellar stock solutions were added to the peptides to reach a final detergent and peptide concentrations of 15 mM and 40 μM, respectively.

CD spectra were acquired on a Chirascan spectropolarimeter (Applied Photophysics Ltd., Leatherhead, UK) between 190 and 260 nm using a 1 mm pathlength quartz cell (Starna, Hainault, UK). Spectra were acquired with 1 nm data intervals, 0.5 s integration time and three scans accumulation. The signal was recorded as milli-degrees at 25 °C.

## 4. Conclusions

In conclusion, the chemical synthesis of an 85 residue EqtII analogue was achieved. By employing microwave-assisted Fmoc SPPS, peptide precursors with over 40 residues were assembled efficiently. After modification of the EqtII(1–46) fragment to the reactive C-terminal thioester, NCL was performed with the A47C mutated EqtII(47–85) peptide. The 1–85 analogue was formed without the formation of any significant side products and isolated via RP-HPLC at high purity. CD spectroscopy indicated that the N-terminal domain of EqtII(1–46) and EqtII(1–85) maintains predominantly an α-helical structure in solution and also in the presence of DPC and SDS micelles. Finally, this work demonstrates the feasibility of assembling the ~20 kDa protein toxin EqtII via chemical means. Once all ligation and desulfurization reactions are optimized, site-specific isotopic labels could then be incorporated to aid in the elucidation of its mechanism of action with membranes via solid-state NMR spectroscopy.

## Supplementary Materials

The following are available online: NMR spectra of the peptides.
