# Supplementary material for: Chemical Synthesis and Characterization of an Equinatoxin II(1–85) Analogue
Source: Molecules. 2017 Mar 30;22(4):559. doi: 10.3390/molecules22040559 (PMC6153748; doi:10.3390/molecules22040559)
Supplement: Supplementary file 1 [file molecules-22-00559-s001.pdf]

Supplementary data

for

Chemical Synthesis and Characterization of an Equinatoxin II (1-85) Analogue

Contents

Figure S1. Solution ^{1}H NMR in DPC micelles pp 1-2.

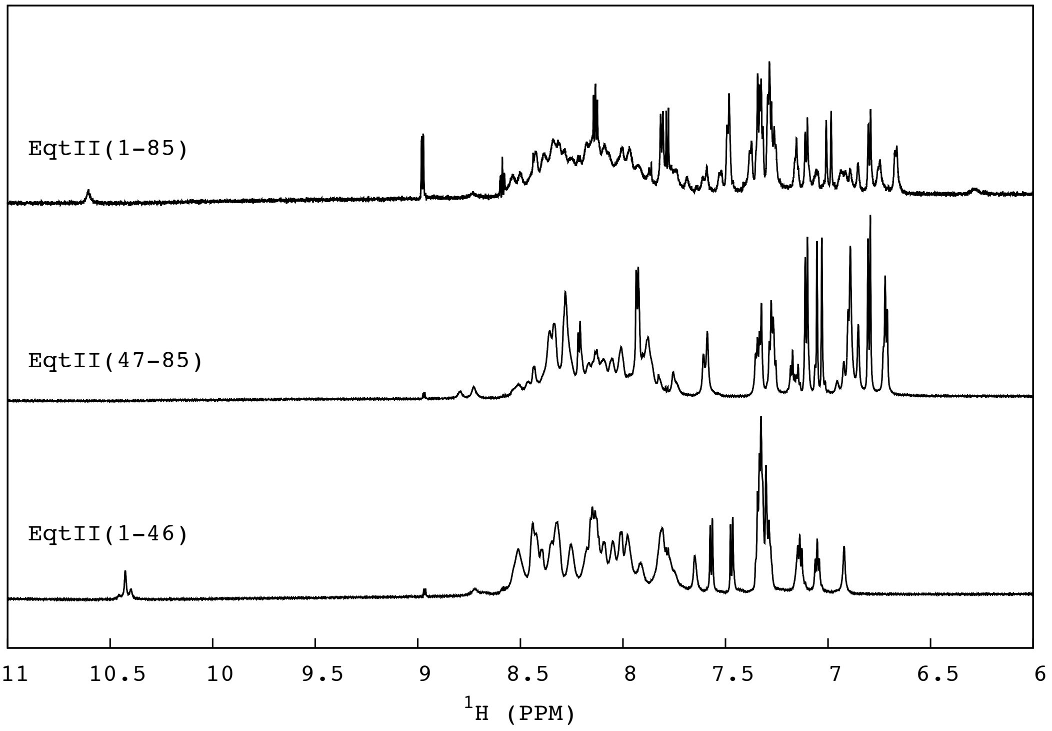

Figure S1. ^{1}H solution NMR spectra of amide region of EqtII(1-46) fragment (bottom), EqtII(47-85) A47C fragment (middle), and EqtII(1-85) fragment (top) in the presence of d_{38}-DPC micelles. NMR samples were prepared by dissolving the dry peptides in 50 mM perdeuterated DPC (d_{38}-DPC, 20 mM phosphate pH 7.4, 50 mM KCl, 0.05 mM 4,4-dimethyl-4-silapentane-1-sulfonic acid (DSS), 10%v D_2O). The concentrations of EqtII(1-46), EqtII(47-85), EqtII(1-85) and d_{38}-DPC were 1 mM, 1 mM, 0.05 mM and 50 mM, respectively.

^{1}H 1D NMR spectra were obtained at 298 K on an 800 MHz Bruker Advance II spectrometer equipped with a 5 mm TCI cryoprobe. Chemical shifts were referenced to DSS at 0 ppm. Data were processed in NMRPipe [1] and plotted using Gnuplot 4.6.

1. Delaglio, F., Grzesiek, S., Vuister, G. W., Zhu, G., Pfeifer, J., Bax, A. NMRPipe: a multidimensional spectral processing system based on UNIX pipes. *J. Biomol. NMR* **1995**, 6, 277–293.
